# Automated Semisupervised Measurement of Optic Nerve Sheath Diameter From CT Following Traumatic Brain Injury

**DOI:** 10.1155/ijbi/9929121

**Published:** 2026-08-05

**Authors:** Emily Wittrup, Alan Kay, Elizabeth Lombard, Jett Rosen, Ethan Schnathorst, Christine Geng, Haoyuan Ma, Erica B. Stein, Kevin R. Ward, Craig Williamson, Kayvan Najarian

**Affiliations:** ^1^ Gilbert S. Omenn Department of Computational Medicine and Bioinformatics, University of Michigan, Ann Arbor, Michigan, USA, umich.edu; ^2^ Department of Radiology, Michigan Medicine, Ann Arbor, Michigan, USA, umich.edu; ^3^ Department of Emergency Medicine, Michigan Medicine, Ann Arbor, Michigan, USA, umich.edu; ^4^ Max Harry Weil Institute for Critical Care Research and Innovation, Michigan Medicine, Ann Arbor, Michigan, USA, umich.edu; ^5^ Division of Neurocritical Care, Department of Neurosurgery, University of Michigan, Ann Arbor, Michigan, USA, umich.edu; ^6^ Michigan Institute for Data and AI in Society (MIDAS), University of Michigan, Ann Arbor, Michigan, USA, umich.edu; ^7^ Center for Data-Driven Drug Development and Treatment Assessment (DATA), University of Michigan, Ann Arbor, Michigan, USA, umich.edu

**Keywords:** artificial intelligence, CT, optic nerve, semisupervised learning, traumatic brain injury

## Abstract

Automated measurement of optic nerve sheath diameter (ONSD) from computed tomography (CT) scans is clinically important, but deep learning methods are often limited by the scarcity of high‐quality, expert‐labeled data given the labor‐intensive nature of manual segmentations. To address this, we present a fully automated modular pipeline combining deep learning and semisupervised learning techniques for ONSD estimation from axial head CT scans. The pipeline features three stages: (1) deep learning‐based slice selection, (2) semisupervised machine learning for optic nerve segmentation utilizing both labeled and unlabeled publicly available data, and (3) geometric and morphological ONSD measurement. When validated on public and internal datasets, our approach generalized better than traditional supervised segmentation methods, achieving an intersection over union (IoU) of 0.561 ± 0.141 and Dice coefficient of 0.707 ± 0.131 on previously unseen data. Slice‐wise measurement accuracy varied based on measurement distance from the ocular globe, yielding mean absolute errors as low as 1.779 and 1.899 mm for the right and left ONSD, respectively. Our findings highlight the potential for semisupervised deep learning to deliver fully automated ONSD measurements and the framework′s adaptability to difficult medical imaging tasks even with limited, low‐quality ground truth for training.

## 1. Introduction

Artificial intelligence (AI) and machine learning (ML) methods hold immense promise in advancing medicine and critical care, including the treatment of traumatic brain injuries (TBIs), which accounted for more than 200,000 hospitalizations in the United States in 2020 [1]. However, these models typically require sizable, expertly labeled ground truth training datasets—which, in the case of small region of interest (ROI) medical segmentation models, are difficult, time‐consuming, and expensive to curate, in addition to being prone to annotator variations [2–6]. To address this challenge, methods such as transfer learning [7], unsupervised learning [8], and semisupervised learning [9] have emerged. Specifically, semisupervised learning harnesses the power of large, unlabeled datasets in combination with smaller labeled ones to enhance model training [2].

Recent TBI research has explored methods to quickly perform initial assessments to determine the best course of clinical care. One critical measure is a patient′s intracranial pressure (ICP), which provides an assessment of injury severity and informs clinical decisions, such as the need for surgical intervention. Monitoring ICP—which may be associated with reduced mortality for severe TBI [10, 11]—is typically performed via an invasive intracranial device (IID); however, this comes with additional risks such as intracranial hemorrhage, dislocation, and device‐associated infection [12–15]. Furthermore, IID insertion is typically only performed at specialized trauma centers and thus significantly limits early precision interventions to victims of TBI in rural areas. Therefore, noninvasive proxies of invasive ICP monitoring via IID have been explored.

In particular, the optic nerve sheath diameter (ONSD)—which is known to be affected by changes in ICP [16]—has shown particular promise. Various imaging modalities including ultrasonography, computed tomography (CT), and magnetic resonance imaging (MRI) have been explored to measure the ONSD as an indicator of elevated ICP [17, 18]. Prior research has primarily focused on ONSD measurements from ultrasonography [19–29]; however, due to variations in device positioning, image quality, and operator variations, there are practical limitations of accurate, automated ONSD measurements from ultrasounds [30]. Alternatively, CT scans are routinely collected for TBI patients, have higher spatial resolution, and are not prone to variations in measurement based on the angle of insonation. Studies have shown agreement between ONSD measurements from ultrasound and CT [31, 32] and recent studies have validated the relationship between manual CT ONSD measurements and ICP in TBI patients [18, 33–37]. When ONSD measurements are made manually from CT, they are still prone to some human variability [38], and the process can be time‐consuming in practice. Therefore, there is a need for a consistent and automatic method of measuring the ONSD from head CTs.

To automate ONSD measurement, image segmentation AI/ML methods have been deployed to segment the optic nerve (ON) from head CT scans [39–46] enabling ONSD measurement and downstream ICP estimation. In particular, convolutional neural networks (CNN) [39, 41–44, 47], transformers [40, 48], state‐space models [49], and multiview networks [50] have been employed to segment the ON from head CT slices—including training techniques such as multiscale supervision [43], transfer learning [42], self‐supervised learning [46], and semisupervised learning [45]—obtaining promising segmentation results. However, existing studies rely on expert segmentation masks and manual identification of relevant axial slices containing the ON for model training. Furthermore, only Lee et al. [41] included an automated ONSD measurement step, which is critical for ICP estimation.

Herein, we propose an automated, three‐stage pipeline for noninvasive automatic ON segmentation and ONSD measurement from axial head CT slices. The specific contributions of this work are as follows:

• The implementation and validation of a cutting‐edge computer‐vision semisupervised learning model for medical image segmentation. This approach is particularly valuable in scenarios where there are limited ground truth segmentations even when those segmentations are low quality. The model is explicitly validated herein on a challenging segmentation task with a small ROI; however, it has broad potential applications across medical domains.
• The proposal of a comprehensive framework to transition seamlessly from raw CT scans to the measurement of ONSD is presented. This pipeline′s modular structure allows for easy adaptation and specialization to specific populations or institutions.
• A detailed assessment of measurement accuracy using an external validation cohort to explore robustness and generalizability of the proposed methodology.

The following article′s methods and results are divided between the pipeline′s three modules that sequentially select relevant axial slices, segment the ON, and measure the ONSD.

## 2. Methods

**Figure 1:**
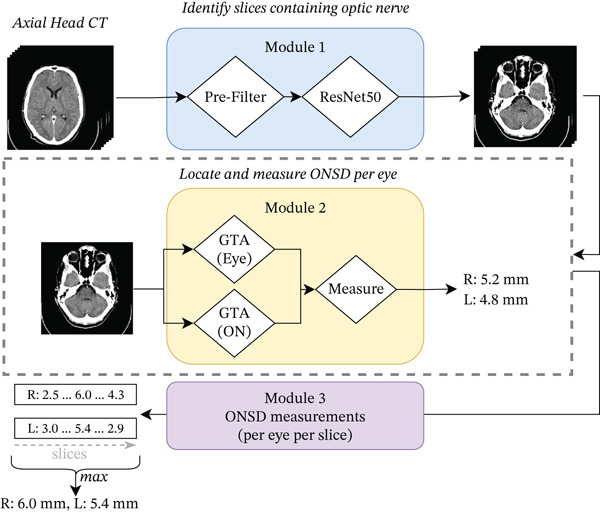
Schematic diagram of automated optic nerve sheath diameter (ONSD) estimation modular pipeline. Module 1 first selects the slices predicted to contain the optic nerve (ON). Module 2 creates eye and ON segmentation masks from which Module 3 measures the ONSD for both the right and left eyes. CT, computed tomography; ResNet, residual network; ON, optic nerve; ONSD, optic nerve sheath diameter; GTA, gentle‐teaching assistant; R, right optic nerve diameter; L, left optic nerve diameter.

Our modular three‐stage framework provides a fully automated approach to estimating the ONSD from axial head CT scans (Figure 1). The first module consists of a deep learning CNN framework for automated axial slice selection from head CT scans that are likely to contain the ON. All previous studies segmenting the ONSD or correlating manual CT ONSD measurements with ICP started with manually preidentified ON slices [18, 33–36]. However, to support full automation for prompt results in a critical care setting, these slices first must be identified from the entire CT volume. To achieve this, we employed a combination of a statistical filter and ResNet50 to accurately identify slices that contain the ON. The slices passing through Module 1 are then passed into Module 2, which generates ON and eye segmentation masks. Due to limited labeled data available for such a task, we utilized a semisupervised Gentle Teaching Assistant (GTA) paradigm to incorporate unlabeled TBI CT slices into the training process. The resulting segmentations are used in Module 3 to create left and right ONSD measurements, which are aggregated within each scan. Each module will be discussed in greater detail in the following sections.

### 2.1. Data

Dataset characteristics, available labels, and role in training each module are outlined in Table 1. Preprocessing was applied to each dataset as described to standardize the image sizes and appearances.

**Table 1:** Imaging characteristics and labels available for included imaging datasets. All values are displayed as counts.

		PDDCA	RSNA	MM
Total	Patients	48	272	96
	CT scans	48	323	208
	Slices	7367	10,790	12,489
Contains ON (slices)	w/ON	203	1136^*α*^	—
	w/out ON	7164	9654^*α*^	—
Segmented ON (slices)	Train/validation	7367	80^*α*^	—
	Test^*β*^	10	9	10
Measured ONSD^*β*^ (scans)		—	—	208
Role in training	Module 1	Filter only	Filter + model	None
	Module 2	Labeled	Unlabeled^*γ*^	None

Abbreviations: CT, computed tomography; MM, Michigan Medicine; ON, optic nerve; ONSD, optic nerve sheath diameter; PDDCA, Public Domain Database for Computational Anatomy; RSNA, Radiological Society of North America.

^*α*^Manual nonexpert annotations.

^*β*^Manual expert annotations.

^*γ*^Except ratio experiments.

#### 2.1.1. Public Domain Database for Computational Anatomy (PDDCA)

The PDDCA dataset contains head CTs from the Radiation Therapy Oncology Group 0522 multi‐institutional clinical trial by Ang et al. [51]. The Version 1.4 of the dataset has 48 CTs from patients with Stage III or IV squamous cell carcinoma together with manual segmentation of anatomical structures including both ONs [52]. The CTs have a slice thickness ranging from 1.25 to 3 mm. All images were zoomed by a factor of 2 using bilinear interpolation while maintaining their original shape. This dataset was used for pipeline training and validation.

#### 2.1.2. Radiological Society of North America (RSNA)

The RSNA published a dataset in 2019 for a challenge titled “Intracranial Hemorrhage Detection” [53, 54]. The dataset contains more than 674,000 head CT slices from patients with acute intracranial hemorrhage.

The slices were rotated 90° such that all scans were oriented with the patient facing the same direction, and the provided rescaling slope and intercept were applied. This dataset was used for pipeline training and validation.

#### 2.1.3. External Validation

An external validation dataset of 123 patients treated at Michigan Medicine for TBIs between January 1, 2017, and June 30, 2022, was curated for this project. Inclusion criteria for this cohort included patients who were greater than 18 years of age and who had an IID placed at Michigan Medicine. All axial CT scans taken within 5 days following the patient′s arrival to the emergency room were included, resulting in 96 unique patients and 208 scans. Detailed characteristics of the patients and scans can be found in Tables B1 and B2, respectively. Scans were acquired with scanners from three manufacturers: General Electric Medical Systems, Siemens, and Neurologica. All slices were resized to 512 × 512 pixels. This dataset was withheld from training of the pipeline and used only during inference as unseen external validation.

### 2.2. Module 1: Slice Selection

The first module selects slices from full axial CT scans that are likely to contain the ON using a combination of deep learning and manual labeling. Although not necessary for ON segmentation, this step helps alleviate the class imbalance presented to Module 2 since the ON is small relative to the full head CT scan.

#### 2.2.1. Label Curation

The project team visually assessed 10,790 unique axial slices from 323 head CT scans from the RSNA dataset and assigned labels specifying whether each slice contained either the right or left ON (Table 1). Labeling was performed by nine researchers under instruction from a board‐certified radiologist. Both the label and reviewer confidence were recorded, and the latter was used to reconcile scans that were labeled by more than one reviewer for improved ground truth annotation.

#### 2.2.2. Statistical Prefilter

Within an axial head CT scan, slices contain distinct structures that can be loosely identified via the standardized Hounsfield unit (HU) values assigned to each pixel by the CT scanner. We first empirically analyzed the HU values to filter out slices that were unlikely to contain the ON.

Standard HU windows such as brain soft tissue, brain bone, and soft tissue were evaluated for discriminative power between slices known to contain the ON versus those that did not contain the ON in the combined PDDCA and RNSA datasets. For each slice, the HU values within each window were summarized by taking the median. In particular, the brain bone window (window level: 600; window width: 1400) showed all ON slices closely clustered around 0 (*μ* = 27.91, *σ* = 10.13) median slice HU, whereas the non‐ON slices had a much wider range of median HU (*μ* = −13.06, *σ* = 119.53) median slice HU (Figure A1); the distributions were significantly different (K‐S: 0.345; *p* < 0.001). Therefore, a filter threshold was introduced such that all slices with median HU values within the brain bone window falling outside of the range −32.85 to 88.68 HU were removed. In both public datasets, applying the filter did not remove any slices labeled as containing the ON but removed 41.93% and 12.65% of non‐ON slices, respectively, for the PDDCA and RSNA datasets (Table 2).

**Table 2:** Module 1 slice selection per dataset. The numbers presented represent the number of axial CT slices that respectively passed the statistical slice filter and were subsequently predicted by Module 1 to contain the optic nerve. All values are displayed as counts.

	Passed slice filter	Slice predicted to contain ON
PDDCA	4278	203
RSNA	9425	1131
MM	10,910	1766

Abbreviations: CT, computed tomography; MM, Michigan Medicine; ON, optic nerve; PDDCA, Public Domain Database for Computational Anatomy; RSNA, Radiological Society of North America.

#### 2.2.3. Deep Learning for Slice Selection

After prefiltering slices, the absolute pixel values from the remaining CT slices from the RSNA dataset were truncated and scaled. Prior to training the slice selection model, each axial slice was augmented with identical slices after (1) histogram equalization and (2) histogram equalization and canny edge detection with lower and upper thresholds of 40 and 150, respectively. Resulting tensors were randomly transformed with elastic transformations. Due to the small sample size, the dataset was randomly shuffled patient‐wise into three training and testing cohorts. For each run, 10% of the training data were sampled as the validation set, stratified by whether the slices contain the ON. Fixed seeds were used across all experiments.

A ResNet50 architecture [55] with ImageNet pretrained weights [56] was chosen for this task given its excellent ability to capture spatial information. ResNet50 consists of a stack of residual blocks that allows for deeper networks without facing the vanishing gradient problem [55]. Each residual block in ResNet50 is a set of three layers consisting of a 1 × 1, 3 × 3, and another 1 × 1 convolution, with each convolutional layer followed by a batch normalization layer and a rectified linear unit (ReLU) [55]. The model was trained for 25 epochs with a learning rate of 0.0001 and a batch size of 16 with the Adam optimizer. Weighted binary cross entropy (BCE) loss with an additional positive class multiplier *γ* was employed during model training:

lx,y=−1N∑n=1Nγ·NnNp·ynlogσxn+1−yn·log1−σxn,

where *N*_*n*_ and *N*_*p*_ represent the number of samples in the positive—those containing the ON—and negative class, respectively. The function *σ* represents the predicted probability that slice *x*_*n*_ contains the ON and *y*_*n*_ is the true binary label. Setting *γ* = 2 doubled the weight on the positive class to further increase sensitivity.

Experiments were performed with and without the additional bias term. Model performance was evaluated via the area under the receiver operator curve (AUC), sensitivity, specificity, and F1‐score. All metrics were averaged over the three splits for each experiment.

### 2.3. Module 2: ON Segmentation

After passing all slices from all datasets through Module 1, the slices predicted to contain the ON were then segmented in a second module using a semisupervised approach to counteract the limited ground truth data available. Two versions of Module 2 were trained: one for ON segmentation and one for eye segmentation; details for the latter model are presented in Appendix D.

#### 2.3.1. Label Curation

In addition to the ground truth eye and ON segmentations provided in the PDDCA dataset, a board‐certified radiologist manually curated a distinct test set for each of the three datasets. Expert segmentations were created from four patient scans from the RSNA dataset (nine total slices) and three patient scans from the PDDCA dataset (10 total slices) to serve as the test set. Of the nine RSNA test slices, four did not contain the ON at all, allowing us to test our model′s specificity. An additional 10 slices were segmented from the Michigan Medicine dataset. Furthermore, to adjust for low integrity segmentations in the PDDCA dataset, a validation set of 20 random slices was manually corrected by the radiologist. All manual segmentations were performed in MicroDicom. Finally, an additional 80 RSNA slices were segmented by two nonexpert members of the study team to use as rough labels for the ratio experiments detailed below.

#### 2.3.2. Semisupervised Model

**Figure 2:**
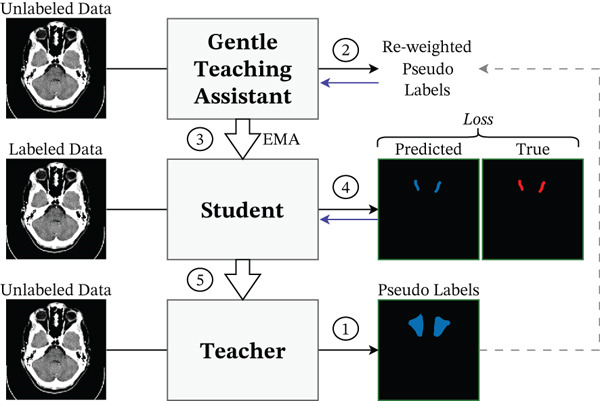
Schematic diagram of the semisupervised model information flow. EMA, exponential moving average.

The GTA architecture was developed by Jin et al. [57] for nonmedical object recognition tasks in computer vision. The model can utilize large amounts of unlabeled data with limited labeled data making it an ideal model for medical applications. This differs from standard semisupervised approaches through the disentangling of the pseudo labels predicted for the unlabeled data and the segmentation model learning from ground truth labels. This is done through the introduction of a third CNN on top of the typical student and teacher framework, dubbed the teaching assistant (Figure 2). Pseudo labels for the unlabeled data are created by the teacher and then subsequently learned by the teaching assistant. The student network is the only model that sees the labeled data directly. The three networks transfer knowledge between them by way of exponential moving average (EMA) [57]. The method results in a prediction mask where the value of each pixel represents the model′s confidence that the pixel is ON as seen in the following equations:

cij=σfxij,y∧ij=1,if cij>0.50,otherwise.

The ON confidence for the pixel at location (*i*, *j*) of image *x* is calculated by applying the sigmoid function, *σ*, to the model′s prediction (*f*(*x*_*i* *j*_)). A threshold is applied on the confidence values to produce the resulting binary segmentation, $\overset{\wedge }{y}$, of the ON. Similar to Module 1, a ResNet50 architecture was used as the backbone that formed the structure of all three models within Module 2. A random grid search was performed to determine the optimal hyperparameter settings for batch size, warmup epochs, learning rate (and associated decay power), and EMA *α*. The ranges for these parameters were 2–12, 1–100, 0.00001–0.01, 0.6–0.9, and 0.01–0.99, respectively.

#### 2.3.3. Training Details

Deviating from the original GTA experimental setup for multiclass segmentation [57], we adapted it for binary segmentation. Gaussian blur and elastic transform data augmentations were applied randomly to 25% of the data to increase generalizability by

specifically addressing low contrast differentiation between ON and background pixels and homogeneity in ON location across scans. Tversky loss was minimized with *α* = 0.7 and *β* = 0.3, and the initial learning rate, batch size, and weight decay for the Adam optimizer were set to 0.003, 3, and 0.0001, respectively, mimicking those in the original GTA experiments [57, 58]. Likewise, the original polynomial learning rate scheduler with a power of 0.9 was applied every epoch. Models trained for 100 epochs, with the first one being a warm‐up epoch (i.e., training all three models in the GTA in a supervised fashion with the labeled data). The model was trained on 173 labeled PDDCA slices and 865 unlabeled RSNA slices; additional ratio experiments were conducted to quantify the performance trend of adding more (or less) labeled and unlabeled data. Focal Tversky with *γ* = 1.33 [59] and BCE loss functions were also explored.

#### 2.3.4. Statistical Testing

To compare segmentation performance against baseline segmentation methods, we treated each test case—combined across all three datasets—as a repeated measure and tested for overall differences in IoU, Dice, and sensitivity using the nonparametric Friedman test (two‐sided; *k* = 4 models). For metrics with a significant Friedman result, we performed pairwise two‐sided Wilcoxon signed‐rank tests with Holm correction for multiple comparisons and report rank‐biserial correlation (*r*) as an effect size.

### 2.4. Module 3: ONSD Measurement

Once ON and eye segmentations were created, a combination of geometry and morphology was applied to automatically measure the ONSD.

#### 2.4.1. Label Curation

For each test set, the following computational method was applied to both the ground truth and predicted segmentation masks to calculate error in ONSD. For the Michigan Medicine external validation dataset, the right and left ONSD for each CT scan was manually measured by a board‐certified radiologist using MicroDicom. The measurements were taken at 3 mm from the ocular globe margin [39]. Three measurements were taken per eye—the variations for which are presented in Table C3—and the average was used for error quantification.

#### 2.4.2. Geometric and Morphologic Measurements

In common practice, ONSD is typically measured at 3 mm posterior to the ocular globe [60]; however, the proposed method allows for the diameter to be collected from any distance posterior to the ocular globe margin given that the ON segmentation exists at that distance.

First, the slice and ON/eye segmentations were enlarged using bilinear interpolation based on the pixel spacing to increase resolution and support more accurate measurements. Then the sagittal midline was determined based on the major axis using the original CT scan, effectively splitting the head into right and left regions. Artifacts on the ON segmentation were removed if they appeared outside of a posterior semicircle with an 8.5‐mm radius centered on the eye segmentation. If no eye segmentation existed for a side on a given slice, or if no ON remained after filtering, no ONSD was collected for that side on that slice.

**Figure 3:**
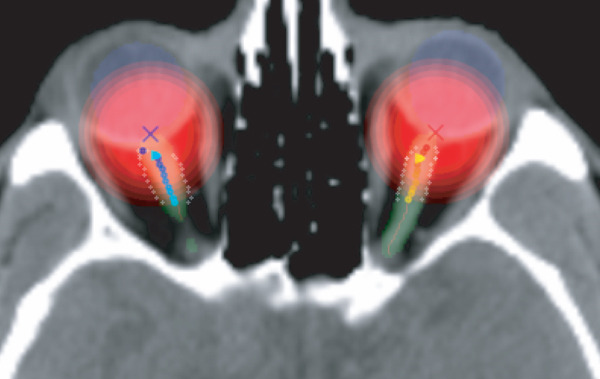
Visualization of the optic nerve sheath diameter (ONSD) measurement from segmentations. The green regions are pixels that represent the generated optic nerve (ON) segmentation, blue regions are eye segmentations, and the overlapping transparent red circles show different distances at which an ONSD was collected posterior to the ocular globe margin, which is marked with an x. Blue markers show the specific distances, and the white plus sign markers show the diameter collected for that point.

The ON object for a given slice and side was then copied to make a skeletonized version producing a midline and a Euclidean distance transform (EDT) version, where each pixel within the object is given the value of the pixel distance to the nearest background pixel. To measure at a certain distance from the posterior globe, the midline pixel nearest that distance is found. The EDT version then provides the distance to the background from that midline pixel, which is doubled to provide the ONSD measurement for that given distance. When available, ONSD measurements were taken every 1 mm from 3 to 12 mm from the posterior globe margin. A visualization of this is presented in Figure 3. Finally, slice‐wise measurements within each CT scan were aggregated to identify the maximum ONSD per eye (Figure 1).

## 3. Results

Results for Module 1 are presented in Table 3. Adding the additional positive bias, *γ*, reliably achieved perfect sensitivity while also having a specificity of 0.989 ± 0.112. For each of the three datasets, PDDCA, RSNA, and Michigan Medicine, the number of slices passing the statistical prefilter and those subsequently predicted to contain the ON are presented in Table 2.

**Table 3:** Module 1 slice selection performance over three randomly selected test sets of three patients each (120 RSNA slices) with and without the positive bias. The reported performance metrics are presented as the mean(std).

Positive bias	Sensitivity	Specificity	AUC	F1‐score
False	0.974 (0.044)	0.996 (0.007)	0.994 (0.010)	0.974 (0.022)
True	1.000 (0.000)	0.989 (0.112)	1.000 (0.001)	0.964 (0.036)

Abbreviation: AUC, area under the receiver operator curve.

For ON segmentation, the validation results from the ratio experiments performed are presented in Table E4. Increasing the ratio of labeled to unlabeled data from 1:1 to 1:3 results in a 0.038 increase in average validation intersection over union (IoU) without having a significant impact on the standard deviation. Likewise, increasing the amount and diversity of labeled data by including additional nonexpert labeled RSNA slices increases performance with diminishing returns with increasing amounts of unlabeled RSNA.

**Figure 4:**
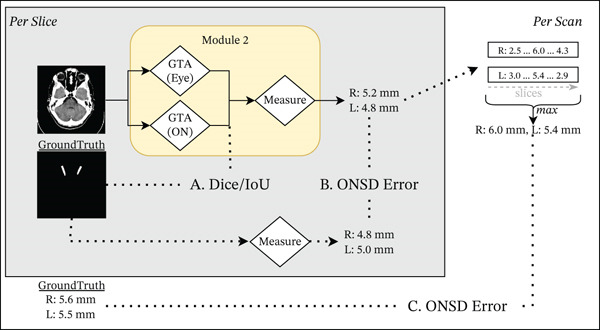
Diagram of quantitative evaluations performed for Module 2. Optic nerve (ON) segmentations from the proposed gentle teaching assistant (GTA) model (and baseline models) are compared with the ground truth segmentations using overlap metrics such as the Dice coefficient and intersection over union (IoU) in Type A error. The optic nerve sheath diameter (ONSD) is measured from both the ground truth segmentation and generated segmentation to calculate Type B error. The measurements are aggregated across slices for the same scan and the max values of the right and left ON are taken. These per‐scan measurements are compared with the manual ONSD measurements for the external validation set in Type C error.

Figure 4 presents a diagram of the quantitative evaluations performed to assess the test results of Modules 2 and 3. In Type A error for Module 2, segmentation overlap metrics (namely IoU, Dice coefficient, and pixel‐wise sensitivity) are calculated between the predicted ON segmentation mask and expert ground truth segmentation mask for each slice in each test set. Within Module 3, the ONSD for each eye is measured on both the predicted and expert ground truth segmentation masks for a single slice, and those measurements are compared quantitatively in Type B error using the following error metrics: mean absolute error (MAE), root mean squared

error (RMSE), mean absolute percentage error (MAPE), and Bland–Altman metrics including bias, 95% limits of agreement (LOA), and proportional bias slope (*β*). Type B error quantifies measurement error introduced by using the predicted segmentation masks over the expert ground truth segmentations both measured automatically via Module 3. After the slices have been aggregated per scan and the maximum value of right and left ONSD across slices has been identified, this slice‐wise ONSD is compared in Type C error using the same error metrics listed above. This comparison occurs between the expert ground truth ONSD labels curated for the Michigan Medicine data and the estimated ONSD from the GTA predicted segmentation masks.

Baseline supervised methods for 2D image segmentation, namely U‐Net, LDN, and ResNet50 (without the GTA paradigm), were all trained on the labeled PDDCA data. Experimental details for these models can be found in Appendix F.

**Figure 5:**
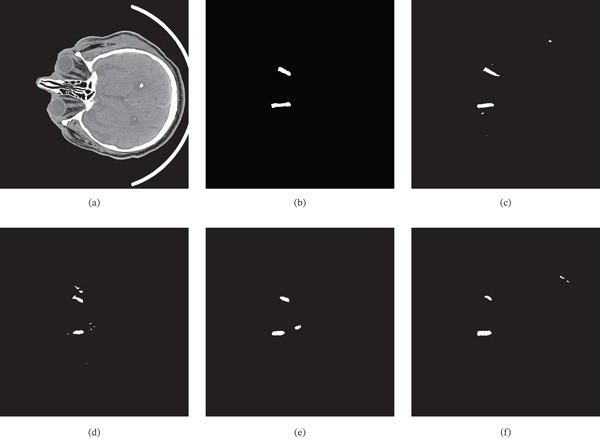
Segmentation mask comparison. (a) Random selected slice taken from the Radiological Society of North America (RSNA) test set and (b) corresponding ground truth optic nerve segmentation. Generated segmentation masks from the (c) Gentle Teaching Assistant (GTA), (d) residual network, (e) U‐Net, and (f) local directional number (LDN) models.

Type A error is presented in Table 4 for the test sets of each of the three datasets. On the non‐TBI PDDCA dataset on which the models were trained, the highest IoU, 0.565 ± 0.122, and Dice, 0.713 ± 0.106, were obtained using the local directional number (LDN) method. For the RNSA TBI dataset, the highest performance was achieved by the proposed GTA approach with an IoU of 0.571 ± 0.0.057 and Dice of 0.725 ± 0.046. This difference can be qualitatively assessed in Figure 5, which presents segmentation masks from each method for a random test sample from the RSNA dataset. Four of the nine RSNA test slices did not contain the ON in the ground truth segmentation

**Table 4:** Type A error segmentation overlap metrics from each model for each test dataset.

Test data	Model	Loss	IoU	Dice	Sensitivity
PDDCA (*N* = 10)	U‐Net	Tversky	0.560 (0.115)	0.711 (0.104)	0.856 (0.121)
	LDN	BCE + Dice	0.565 (0.122)	0.713 (0.106)	0.843 (0.127)
	ResNet	Tversky	0.531 (0.138)	0.682 (0.131)	0.596 (0.110)
	GTA	Tversky	0.472 (0.203)	0.609 (0.234)	0.509 (0.225)
RSNA (*N* = 5)	U‐Net	Tversky	0.504 (0.143)	0.657 (0.142)	0.650 (0.219)
	LDN	BCE + Dice	0.350 (0.193)	0.482 (0.254)	0.541 (0.307)
	ResNet	Tversky	0.447 (0.138)	0.606 (0.122)	0.549 (0.158)
	GTA	Tversky	0.571 (0.057)	0.725 (0.046)	0.731 (0.068)
MM (*N* = 10)	U‐Net	Tversky	0.473 (0.138)	0.629 (0.139)	0.677 (0.207)
	LDN	BCE + Dice	0.278 (0.170)	0.408 (0.207)	0.410 (0.275)
	ResNet	Tversky	0.429 (0.156)	0.585 (0.146)	0.551 (0.171)
	GTA	Tversky	0.561 (0.141)	0.707 (0.131)	0.690 (0.137)
Total (*N* = 25)	U‐Net	Tversky	0.514 (0.136)	0.667 (0.132)	0.743 (0.203)
	LDN	BCE + Dice	0.407 (0.205)	0.545 (0.233)	0.609 (0.307)
	ResNet	Tversky	0.473 (0.153)	0.628 (0.143)	0.568 (0.148)
	GTA	Tversky	0.527 (0.165)	0.671 (0.179)	0.626 (0.195)

Abbreviations: BCE, binary cross entropy; GTA, Gentle Teaching Assistant; IoU, intersection over union; LDN, local directional number method; MM, Michigan Medicine; PDDCA, Public Domain Database for Computational Anatomy; RSNA, Radiological Society of North America.

and were therefore excluded from Table 4; however, the proposed GTA approach had a pixel‐wise specificity of 0.999 ± 0.000 on those slices. Our semisupervised approach was the only model that was able to incorporate unlabeled slices from this dataset into training. Finally, on the Michigan Medicine TBI data, which was withheld from all models during training, the proposed GTA approach generalized better than all baseline methods with an IoU of 0.561 ± 0.141 and Dice of 0.707 ± 0.131.

Across the combined evaluation set (*n* = 25) paired cases, Friedman *χ* ^2^ test returned statistically significant result for IoU (*χ* ^2^ = 9.624, *p* = 0.022), Dice (*χ* ^2^ = 9.624, *p* = 0.022), and sensitivity (*χ* ^2^ = 16.920, *p* = 0.001); however, post hoc Wilcoxon tests with Holm correction indicated only a significant difference in sensitivity between U‐Net and ResNet50 (*χ* ^2^ = 0.809, *p* = 0.001).

**Figure 6:**
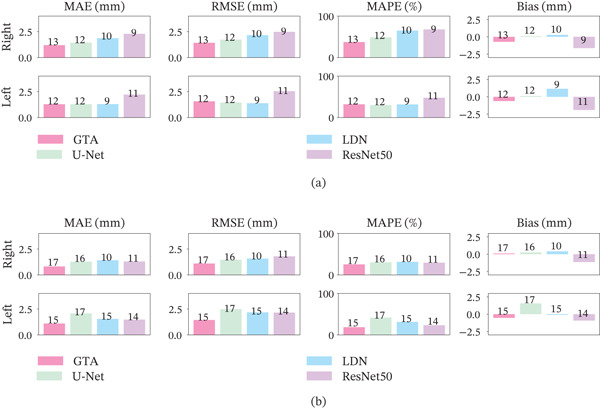
Type B error results for the test set combined across all datasets comparing the optic nerve sheath diameter (ONSD) measurements taken from the ground truth segmentation mask and the generated segmentation mask from each segmentation model. Error is calculated using the measurements from the proposed pipeline taken at (a) 3 and (b) 6 mm from the orbital globe margin, filling with 1‐mm subsequent diameter measurement if missing (i.e., 4 and 7 mm, respectively). The numbers printed on each bar represent the number of test slices for which the ONSD could be calculated at that distance from both the ground truth and generated segmentation masks. MAE, mean absolute error; RMSE, root mean squared error; MAPE, mean absolute percentage error; GTA, gentle teaching assistant; LDN, local directional number.

Type B error is presented in Figure 6 for measurements taken both 3 and 6 mm from the posterior ocular globe margin—the 3‐mm measurement was often missing from the predicted ON segmentation due to edge effects. When either a 3‐ or 6‐mm measurement was missing, it was filled with the value from one subsequent distance (i.e., 4 or 7 mm, respectively) if available. As shown in Figure 6, the proposed GTA method tended to have lower error rates compared with the baseline models at both distances. At 3 mm, the GTA method achieved a right and left MAE of 1.198 and 1.284 mm, RMSE of 1.425 and 1.561 mm, MAPE of 37.289% and 31.804%, and bias of 0.713 and −0.612 mm, respectively. Likewise, at 6 mm, the proposed method had a right and left MAE of 0.817 and 1.093 mm, RMSE of 1.096 and 1.437 mm, MAPE of 25.358% and 18.608%, and bias of 0.095 and −0.525 mm, respectively.

Finally, for the Michigan Medicine external validation set, Type C error between the expert manual ONSD measurements and the automated estimates from the pipeline are presented in Table 5. Six of the 208 scans could not be automatically processed through the pipeline, and two scans did not have a ground truth left ON measurement. At least one right and left 3‐mm (or 4‐mm) ONSD measurement was available for 139 and 127 scans for the right and left side, respectively. Likewise, when moving out to 6 mm, 174 scans had ONSD measurements available. At 3 mm, the left eye was better estimated, whereas at 6 mm the right eye was; however, moving from 3 to 6 mm reduced the error for both eyes (Table 5). The error trends for each metric without subsequent filling are presented in Figure H2. The best performance—compared with the ground truth measurements—was obtained at 6 mm from the ocular globe margin with a MAE of 1.779 and 1.899 mm for the right and left eye, respectively.

**Table 5:** Type C error results comparing the error between the ground truth optic nerve sheath diameter (ONSD) measured by an expert radiologist and the scan‐wise maximum estimated ONSD measurement automatically calculated using the proposed pipeline for the Michigan Medicine external validation cohort. Error is calculated using the measurements taken at 3 and 6 mm from the orbital globe margin, filling with 1 mm subsequent diameter measurement if missing (i.e., 4 and 7 mm, respectively).

Distance	Side	Scans (*N*)	MAE (mm)	RMSE (mm)	MAPE (%)	Bias (mm)	LoA (mm)	*β* (mm/mm)
3 mm	Right	139	2.804	3.100	45.119	−2.780	[−5.477, −0.082]	0.302 ^∗^
	Left	127	2.690	3.060	46.911	−2.538	[−5.902, 0.825]	0.991 ^∗∗∗^
6 mm	Right	174	1.779	2.289	28.580	−1.432	[−4.942, 2.079]	0.983 ^∗∗∗^
	Left	174	1.899	2.353	33.248	−0.644	[−5.093, 3.806]	1.421 ^∗∗∗^

Abbreviations: LoA, 95% limits of agreement; MAE, mean absolute error; MAPE, mean absolute percentage error; RMSE, root mean squared error.

^∗^Proportional bias slope significant to *p* < 0.05.

^∗∗∗^Proportional bias slope significant to *p* < 0.001.

## 4. Discussion

Even with a limited amount of labeled data for training, Module 1 achieved perfect sensitivity when the positive bias was applied—further prioritizing sensitivity during training—while maintaining high specificity (Table 2). The resulting model reduced the class imbalance presented to Module 2 without removing any slices that were likely to contain the ON. Since axial slices located in the ocular region have distinctive and consistent anatomy compared with those in other regions of the head, the model is able to robustly distinguish between them, providing a critical, novel addition to fully automated pipelines.

Next, for ON segmentation, Module 2 was trained on the non‐TBI PDDCA head CT axial slices and the corresponding ON segmentation masks. Given the relatively small size of the ON and the similarity in appearance to the surrounding muscles, segmenting the ON is a difficult task for any AI/ML model. The semisupervised GTA model, which was augmented with unlabeled RSNA TBI data, generalized better to the TBI test sets (Table 4). Furthermore, as shown qualitatively in Figure 5, GTA creates ON segmentation masks that more closely mimic the ground truth. Although previous deep learning‐based approaches for ONSD segmentation from CT scans have reported superior results, Ranjbarzadeh et al. [39] achieved a Dice of 0.877 with their LDN preprocessing method; Li et al. [40] a Dice of 0.839 with their global features extraction method; Lee et al. [41] a Dice of 0.6387 using a conventional CNN—all have used high‐integrity, hand‐annotated datasets for training and validation.

Given the previously mentioned difficulty in curating such a dataset in medicine, the focus of this model was to utilize only limited, publicly available data during training as a prototype for other medical segmentation applications. Previous ON segmentation methods evaluated on the PDDCA dataset specifically had left/right Dice values around 0.66 ± 0.09/0.650 ± 0.090 [47, 48], and 0.793/0.805 [50], which more closely match the results presented herein. Computational comparison of the provided PDDCA ON segmentation masks with the expert corrected versions illustrates a large quality gap with an IoU of only 0.269 ± 0.151, Dice coefficient of 0.401 ± 0.198, and sensitivity of 0.561 ± 0.0333 (Table G5). This is consistent with the findings of Li et al. [40], which showed markedly worse segmentation performance on the PDDCA dataset—left/right ON Dice 0.751 ± 0.040 and 0.782 ± 0.060—than expert curated alternatives (0.835 ± 0.056). In addition, Francis et al.′s [46] model achieved worse Dice segmentation performance of the ONs when trained on the PDDCA data as opposed to other datasets. Among the segmentation models compared herein, all performed worse on the expertly corrected PDDCA validation mask than on the original mask; however, GTA showed a lower drop in performance (Table G5). Taken altogether, these results suggest that the PDDCA ON segmentation masks are low quality relative to expert annotations, which likely accounts for some of the performance decrease over previous methods. In addition, GTA is more robust to low‐quality ground truth data during training than alternative supervised models. Overall, these results illustrate the potential of semisupervised learning in medical applications, specifically when labeled data are limited or low quality, and the ROI is relatively small.

In addition to segmentation performance improvements over baseline segmentation models, GTA′s predicted segmentation masks produce ONSD measurements that are generally closer to those obtained using the same method from the expert corrected ON segmentation masks from the test sets of all three datasets suggesting higher‐integrity predicted segmentation masks (Type B error; Figure 6).

Finally, when the entire pipeline was applied to the external validation dataset to generate estimated ONSD measurements, there was magnitude‐dependent error, but the MAE was as low as 1.779 for the right eye (Type C error; Table 5). The GTA‐generated ON segmentation masks did not reliably capture the edges of the ON, particularly close to the eye (i.e., 3 mm); however, when moving out to 6 mm from the ocular globe margin, a more accurate ONSD measurement could be made from the middle of the segmentation (Figure H2). It is estimated that in healthy individuals, ONSD roughly ranges from 4 to 6 mm [60], while in patients with Glasgow Coma Scale (GCS), the range 2–8 can increase ONSDs to on average 6.4 ± 1.0 mm [61]. Therefore, current automated ONSD measurements have roughly a 24%–32% error when compared with the measurements taken from a single expert clinician. This level of error and negative systematic bias preclude direct clinical applicability of this model for ICP screening decisions.

Lee et al. [41] reported manual ONSD measurements from their model‐predicted segmentation masks on the first two slices per eye (slice thickness of 3 mm) for which the ON appeared. They split their results by sex and age groups with their best performance obtained for the right eye of males aged 19–64 using only the first slice on which the ON appeared (ground truth of 1.2 ± 0.7 mm and predicted ONSD of 1.4 ± 0.3 mm) [41]. No attempt was made to obtain a representative ONSD estimate per eye per scan across all ON slices. The error rates for AI/ML methods measuring ONSD from ultrasound generally report superior performance [62]; however, are still prone to the previously mentioned limitations of the differences in angle of insonation which persist through the training and validation sets of these studies.

Despite the seemingly inferior performance of the presented pipeline explained by the aforementioned factors, it has specific advantages that make it worth consideration. Namely, the ability of our semisupervised segmentation model to generalize to unseen TBI data even when trained on a limited dataset of non‐TBI scans with low integrity labels suggests the potential of the method to reduce the necessity of time‐consuming expert annotations for similar tasks, speeding up development processes. In addition, our pipeline is fully automated from original CT scan to ONSD measure, which sets it apart from previous work with the additions of Modules 1 and 3. This modular approach is designed to be easily implementable and fine‐tuned for specific institutions or with additional datasets or higher quality segmentation masks. Given the results presented in Table E4, it is hypothesized that either additional labeled data or higher integrity labels will further increase the performance of the model.

In addition to the utilization of the limited, low integrity PDDCA segmentation masks in Module 2 training by design as discussed above, an additional limitation to the existing method is difficulty segmenting the ON close to the eye resulting in missing 3‐mm automated measurements. Increasing the integrity and amount of training data is expected to increase the performance of the model, which could be augmented with additional postprocessing steps to enhance the ON segmentation masks at closer distances to the ocular globe. Expert ONSD measurements were taken at 3 mm behind the ocular globe; however, given the resolution of the visualization software and that all measurements were taken by one annotator, there could be variations among the ground truth ONSD label integrity resulting in the heightened performance when GTA ONSD measures were taken at 6 mm. Additionally, alternate pretrained models could be considered for initialization of the GTA model—specifically those pretrained on medical images. Additional validation should test the model′s generalizability to less severe cohorts with a greater diversity of injuries. Finally, given the relatively small volume of the ON, the slice thickness of the CT scans may limit accurate measurement of the true maximum ONSD at a specific distance. Therefore, additional development and validation including validation of the relationship between GTA ONSD and ICP would be necessary prior to clinical deployment.

## 5. Conclusion

In summary, these results demonstrate the potential efficacy of the proposed automated, modular, semisupervised learning pipeline for ONSD measurement to inform TBI patient care. Beyond this specific application, this method has potential for other medical imaging applications where large amounts of expertly labeled data are not available.

**Nomenclature**
AI: artificial intelligence
AUC: area under the receiver operator curve
BCE: binary cross entropy
CNN: convolutional neural network
CT: computed tomography
EDT: Euclidean distance transform
EMA: exponential moving average
GCS: Glasgow Coma Scale
GTA: Gentle Teaching Assistant
HU: Hounsfield unit
ICP: intracranial pressure
IID: invasive intracranial device
IoU: intersection over union
LDN: local directional number
MAE: mean absolute error
MAPE: mean absolute percentage error
ML: machine learning
ON: optic nerve
ONSD: optic nerve sheath diameter
PDDCA: Public Domain Database for Computational Anatomy
ReLU: rectified linear unit
RMSE: root mean squared error
ROI: region of interest
RSNA: Radiological Society of North America
TBI: traumatic brain injury

## Author Contributions

Emily Wittrup was responsible for study design, algorithm development, formal analysis, validation, interpretation and visualization of results, writing the original draft, project administration and contributed to the conceptualization, data curation, and funding acquisition. Alan Kay was responsible for the development of the ONSD measurement algorithm, contributed to the methodology, software development and formal analysis of the segmentation model, data curation, and writing the original draft. Elizabeth Lombard contributed to the methodology and software development of the segmentation model, and writing the original draft. Jett Rosen contributed to the software development of Module 1 and the baseline segmentation models, formal analysis, data curation, and writing the original draft. Ethan Schnathorst contributed to the data curation, methodology, software development, formal analysis for Module 1, and writing the original draft. Christine Geng contributed to the methodology for Module 1 and reviewing and editing the manuscript. Haoyuan Ma contributed to the methodology and software development of Module 1. Erica B. Stein was responsible for funding acquisition, manual labeling and segmentation of all validation and test data, and contributed to both conceptualization and reviewing and editing the manuscript. Kevin R. Ward was responsible for curating the external validation cohort, conceptualization of the project, and reviewing and editing the manuscript. Craig Williamson contributed to the conceptualization of the project, funding acquisition, curation of interreviewer measurements, and reviewed and edited the manuscript. Kayvan Najarian contributed to the conceptualization, funding acquisition, study design, methodology, and provided supervision of the project.

## Funding

This study was supported by Joyce and Don Massey Family Foundation.

## Disclosure

All authors read and approved the final manuscript.

## Ethics Statement

The data collected from Michigan Medicine were used in our retrospective study. Our study obtained approval from the Institutional Review Boards of the University of Michigan Medical School (HUM00092309). Written informed consent from patients has been waived by the University of Michigan Institutional Review Board since this study involves no more than minimal risk to the subjects involved. All methods were performed in accordance with the relevant guidelines and regulations.

## Consent

The authors have nothing to report.

## Conflicts of Interest

C.W. is an unaffiliated neurotrauma consultant with the National Football League. The other authors declare no conflicts of interest.

## Supporting information


**Supporting Information** Additional supporting information can be found online in the Supporting Information section. Additional methods and results are provided in the attached Supporting Appendix.

## Data Availability

The PDDCA (Version 1.4.1; https://www.imagenglab.com/newsite/pddca/) and RSNA (https://www.kaggle.com/c/rsna-intracranial-hemorrhage-detection/overview) datasets are publicly available. The external validation data analyzed during the current study were collected at Michigan Medicine. The University of Michigan′s Innovation Partnerships unit will handle potential charges/arrangements of the use of data, models, and code by external entities, using such methods as material transfer agreements. Please contact innovationpartnerships@umich.edu for data inquiries.
