# Supplementary material for: Automated Semisupervised Measurement of Optic Nerve Sheath Diameter From CT Following Traumatic Brain Injury
Source: Int J Biomed Imaging. 2026 Aug 5;2026:9929121. doi: 10.1155/ijbi/9929121 (PMC13442929; doi:10.1155/ijbi/9929121)
Supplement: Supplementary file 1 [file IJBI-2026-9929121-s001.pdf]

Appendix A Module 1 Statistical Slice Filter

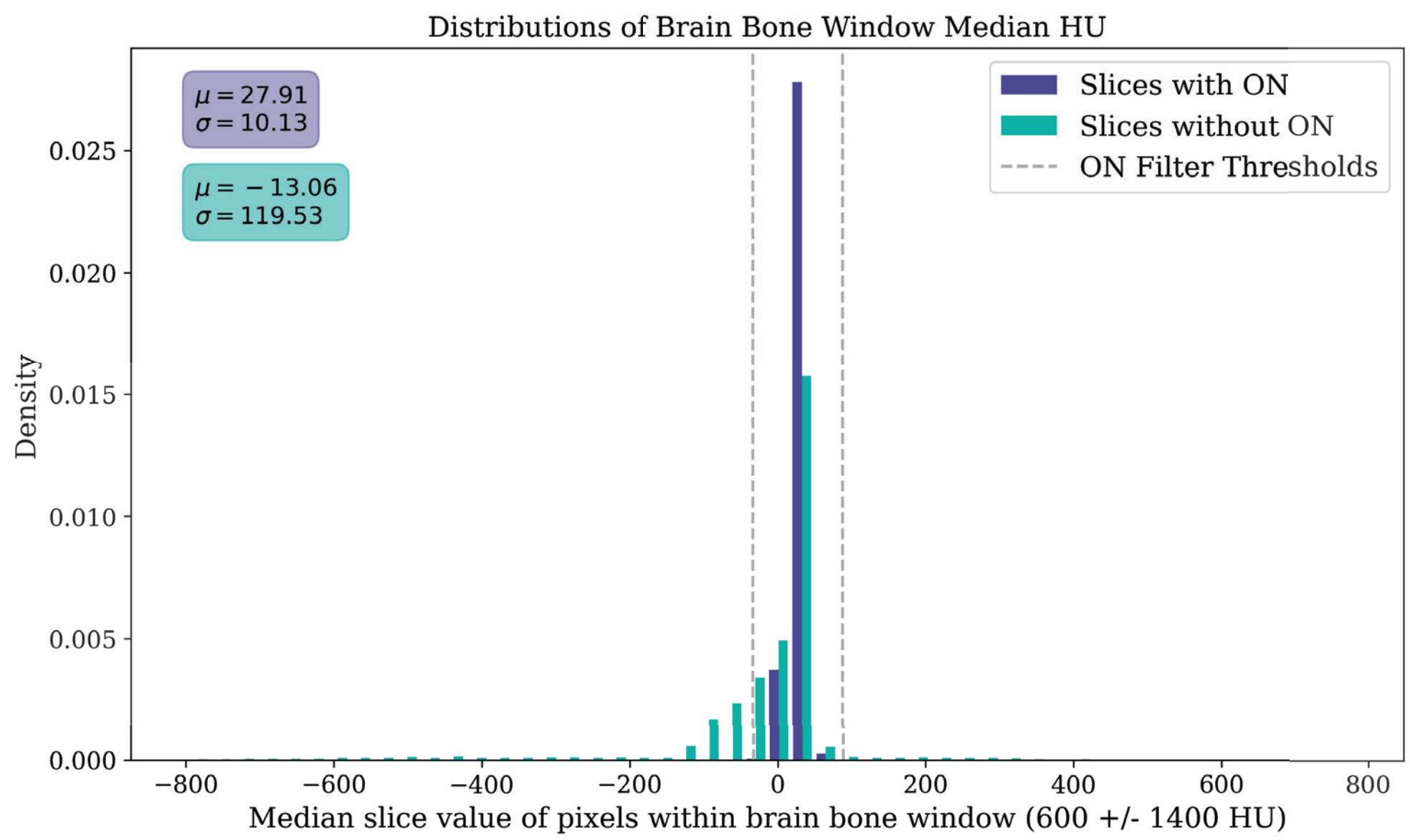

Fig. A1: Module 1 statistical filter visualized over slice-wise brain bone window median Hounsfield units (HU) distributions for slices labeled as containing the optic nerve (ON) and not containing the ON across the RSNA and PDDCA datasets collectively. Lower and upper median value threshold for filtering out slices without the ON were set at -32.85 and 88.68 HU respectively and are visualized as dotted grey lines. *PDDCA: Public Domain Database for Computational Anatomy; RSNA: Radiological Society of North America*

Appendix B External Validation Patient and Scan Characteristics

Table B1: Patient demographics and treatment information from the Michigan Medicine dataset. All values are presented as mean (standard deviation) or median [interquartile range] unless otherwise specified. *GCS: Glasgow Coma Scale; ICP: intracranial pressure; IPPM: intraparenchymal pressure; PbtO2: brain tissue oxygen; JVB: jugular venous bulb catheter*

**Patients** (n)	96
**Age** (years)	46.75 (19.44)
**Male** (n)	67 (69.79%)
**GCS**	3 [3, 7]
**Arrival** (n)	
Scene	58 (60.42%)
Referral	38 (39.58%)
**Therapy** (n)	
ICP Monitor	96 (100.00%)
Brain Operation	31 (32.29%)
Ventilator	65 (67.71%)
IPP Monitor	44 (45.83%)
PbtO2 Monitor	18 (18.75%)
JVB	96 (100.00%)

Table B2: Computed Tomography (CT) scan characteristics from the Michigan Medicine dataset. All values are presented as mean (standard deviation) or median [interquartile range] unless otherwise specified. *ONSD: optic nerve sheath diameter; IPPM: intraparenchymal pressure; PbtO2: brain tissue oxygen*

**Scans** (n)	208
**Scans per patient**	2 [1, 3]
**Left ONSD** (mm)	5.72 (0.87)
**Right ONSD** (mm)	6.10 (0.87)
**Time to scan** (minutes)	582 [45, 2303]
**Scans Post-Therapy**	
Brain Operation	43 (20.67%)
IPP Monitor	37 (17.79%)
PbtO2 Monitor	19 (9.13%)

Appendix C Intra-Annotator Variations in Ground Truth Measurements of Optic Nerve Sheath Diameter

Table C3: Variations among scan-level ground truth measurements of optic nerve sheath diameter (ONSD) for the Michigan Medicine data which were performed three times per scan by the same expert radiologist. *MAE: mean absolute error; RMSE: root mean squared error; r: correlation; MAPE: mean absolute percentage error*

	**Scans**	**MAE**	**RMSE**	**r**	**MAPE**
Right	200	0.155	0.191	0.975	2.788
Left	202	0.176	0.219	0.969	2.973

Appendix D Eye Segmentations Model Training Details

To create eye segmentations for the axial CT slices to aid in measuring the ONSD from the ON segmentations, a separate instance of GTA was trained. Fifty-five axial slices from the PDDCA and RSNA datasets were manually segmented by the study team. These labeled images, in addition to 800 unlabeled axial slices passing Module 1, were used in training a GTA model with the same ResNet50 backbone as the ON segmentation version. Twelve warm-up supervised only epochs were performed on all three models within the GTA. Then the model was trained for 18 epochs achieving its minimum Tversky loss (with a=0.3 and b=0.7) at 29 epochs. A batch size of 3 was used and elastic and gaussian blur data augmentations were randomly employed during training as in the ON model. A polynomial scheduler with a power of 0.9 was applied every epoch and the Adam optimizer with initial learning rate of 0.001 and weight decay of 0.0001 were utilized. The EMA decay was set to 0.99.

Appendix E Module 2 Semi-Supervised Ratio Experiment Results

Table E4: Module 2 validation mean intersection over union (IoU) obtained using different ratios of labeled and unlabeled data with the Gentle Teaching Assistant (GTA).

		Unlabeled		
		173	346	865
Labeled	173	0.365(0.178)	0.376(0.152)	0.403(0.186)
	253	0.446(0.190)	0.417(0.188)	0.406(0.162)

Appendix F Segmentation Baseline Model Training Details

F.1 ResNet50

The ResNet50 baseline segmentation model was trained for 100 epochs with the lowest validation IoU occurring at epoch 45. It was trained on 173 of the labeled PDDCA images. Tversky Loss with $\alpha = 0.6$ and $\beta = 0.4$ was used in training. All the other training details were consistent with the GTA as described in Section 2.3.3.

F.2 U-Net

Different loss functions including boundary loss, focal Tversky with $\gamma = 1.33$[58], Tversky, and BCE were evaluated to optimize the U-Net for the ON segmentation task. All of the U-Net models were trained on the 173 PDDCA image training dataset. Each model was trained with the Adam optimizer with no weight decay, and an initial learning rate of 0.001 decreased to 0.0001 after 25 epochs. All models were trained for 100 epochs and the best epoch based on validation IoU was selected.

The Tversky and Focal Tversky models α and β settings were optimized. The results for both Tversky loss and Focal Tversky loss aligned with those of Salehi et al. in their original Tversky loss paper, where $\alpha = 0.3$ and $\beta = 0.7$ produced the best results [62].

F.3 Local Directional Number

The LDN algorithm was implemented as described by Ranjbarzadeh et al. [39] and then trained the model using BCE with Dice loss, providing both the original image and the LDN form of the image to the model. The other training details are consistent with Section F.2.

Appendix G Validation Results On Original and Corrected Segmentations

Table G5: Overlap validation results from the PDDCA dataset using the original segmentation and the manual expert segmentations. *PDDCA: Public Domain Database for Computational Anatomy; LDN: Local Directional Number method; GTA: Gentle Teaching Assistant; BCE: binary cross entropy; IoU: intersection over union*

	**Model**	**Loss**	**IoU**	**Dice**	**Sensitivity**
Original	U-Net	Tversky	0.473(0.241)	0.601(0.258)	0.711(0.288)
	LDN	BCE + Dice	0.464(0.247)	0.591(0.261)	0.736(0.303)
	ResNet50	Tversky	0.492(0.179)	0.637(0.192)	0.630(0.219)
	GTA	Tversky	0.423(0.234)	0.554(0.251)	0.546(0.290)
Expert	U-Net	Tversky	0.294(0.135)	0.438(0.166)	0.628(0.286)
	LDN	BCE + Dice	0.291(0.134)	0.435(0.160)	0.667(0.283)
	ResNet50	Tversky	0.366(0.162)	0.514(0.187)	0.609(0.294)
	GTA	Tversky	0.403(0.181)	0.548(0.205)	0.600(0.278)
Original vs. Expert			0.269(0.151)	0.401(0.198)	0.561(0.333)

Appendix H External Validation Scan-Wise Error by Distance

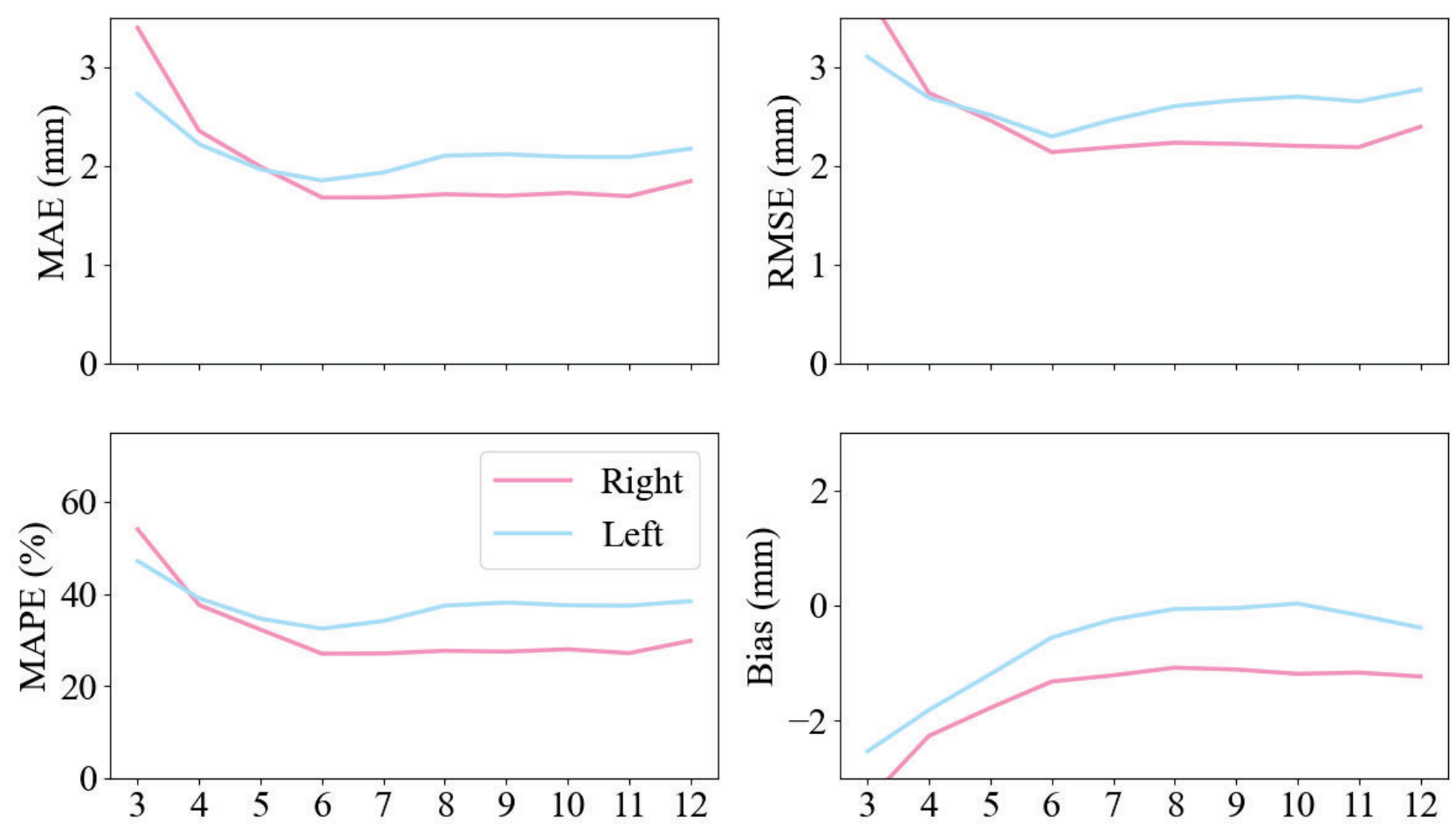

Fig. H2: Error C results comparing the ground truth optic nerve sheath diameter (ONSD) measured by an expert radiologist and the scan-wise maximum estimated ONSD measurement automatically calculated using the proposed pipeline for the Michigan Medicine cohort. Measurements from the proposed method's segmentation mask are taken at every 1mm from 3mm to 12mm behind the ocular globe margin. *MAE: mean absolute error; RMSE: root mean squared error; MAPE: mean absolute percentage error*
